# Operational Stability and Treatment Efficiency of a Reverse Osmosis Plant Treating Highly Mineralized Water: Results of a Four-Year Follow-Up Study

**DOI:** 10.3390/membranes16090292

**Published:** 2026-08-31

**Authors:** Smail Es Sellami, Taleb Abdeslam, Mohammed El Hachoumi, Badr El Fathi

**Affiliations:** Laboratory of Process Engineering and Environment (LGPE), Faculty of Science and Technology of Mohammedia (FSTM), Hassan II University of Casablanca, Mohammedia 28806, Moroccomed.elhachoumi@gmail.com (M.E.H.); badr.elfathi@gmail.com (B.E.F.)

**Keywords:** reverse osmosis, statistical analysis, conductivity, Python

## Abstract

This study evaluates the long-term operational performance of a full-scale reverse osmosis desalination plant treating highly mineralized water under real industrial operating conditions. Unlike conventional laboratory or pilot-scale investigations, the analysis is based on four years of operational data (2020–2024), providing a representative assessment of membrane system behavior under variable environmental and hydraulic conditions. Statistical analyses including descriptive statistics, time-series analysis, Pearson correlation, and inter-annual comparisons were performed using Python-based data-processing tools. An Operational Stability Index was also introduced to quantify process consistency over time. The results demonstrate stable desalination performance, with salt rejection exceeding 90%. Operational monitoring indicated effective pretreatment conditions, low SDI values, and controlled membrane fouling under long-term operation. Differential pressure evolution and cleaning-in-place performance further confirmed the effectiveness of the implemented fouling mitigation strategy. Membrane autopsy investigations revealed the coexistence of biological fouling, silica scaling, and localized oxidative degradation, highlighting the importance of integrated pretreatment and operational control. The findings demonstrate the value of long-term industrial monitoring for understanding membrane performance, fouling behavior, and operational stability in full-scale RO systems. This approach supports data-driven operational optimization and may contribute to the future development of predictive monitoring and intelligent desalination-management frameworks as a Digital Twin.

## 1. Introduction

In light of the gradual depletion of water resources and the water stress affecting semi-arid regions in Morocco, the need for drinking water has become increasingly critical, especially in rural areas. In fact, the high salinity of these resources makes water treatment an unavoidable solution to ensure a continuous supply of drinking water that meets current quality standards.

Reverse osmosis treatment has become nearly ubiquitous, particularly in rural areas, through the installation of integrated systems comprising pretreatment and specific treatment to reduce the salinity of water resources and subsequently meet the needs of the target population. Reverse osmosis is widely recognized for its high efficiency in removing dissolved salts and ensuring the safety of drinking water [1,2,3].

In this study, primary importance was placed on data collection over a period spanning four years from 2020 to 2024, in contrast to recent studies that rely on short-term laboratory results. Unlike laboratory experiments or short-term pilot studies, long-term monitoring allows for the assessment of operational stability, process robustness, and performance variability under fluctuating environmental and operating conditions [1].

Thus, optimization of the performance of the reverse osmosis membranes is a priority given the high operating costs, which include significant expenses for reagents and energy. Admittedly, in Morocco, there are several operational constraints, particularly in terms of technical expertise and know-how, which necessitate the implementation of a strategy based specifically on the principles of digitalization and remote management, while reducing the need for on-site intervention.

Despite the extensive literature on reverse osmosis desalination, most existing studies remain limited to short-term laboratory experiments or pilot-scale investigations conducted under controlled conditions. Long-term industrial evaluations under realistic operating environments remain relatively scarce, particularly in semi-arid regions exposed to progressive salinity increases and hydrological stress. Moreover, many previous investigations focus primarily on membrane rejection performance without integrating operational indicators associated with fouling dynamics, process stability, and hydraulic resilience. Consequently, there remains a critical need for long-term industrial studies capable of linking hydrochemical variability, pretreatment efficiency, and membrane operational stability under real field conditions.

## 2. Materials and Methods

### 2.1. Process Description

The facility investigated in this study is a full-scale reverse osmosis (RO) demineralization plant operated by ONEE–Branche Eau in Khénifra, Morocco. The plant treats highly mineralized surface water under semi-arid climatic conditions and has a nominal production capacity of approximately 30,000 m^3^/day. The treatment line combines conventional physicochemical pretreatment with membrane-based desalination processes in order to ensure stable drinking-water production under fluctuating raw-water quality conditions.

The pretreatment train consists of coagulation–flocculation using ferric chloride or aluminum sulfate, lamellar settling, dual-media filtration (sand/pumice), cartridge microfiltration (5 µm), sodium metabisulfite dosing for dechlorination, sulfuric acid injection for scaling control, and antiscalant dosing. These pretreatment barriers are essential for limiting particulate, organic, and biological fouling prior to reverse osmosis operation. Previous studies demonstrated that efficient pretreatment significantly improves the membrane lifespan and operational stability in full-scale desalination systems.

The reverse osmosis system operates with spiral-wound polyamide composite membranes installed in a two-stage configuration. Two membrane technologies are currently used at the facility. Each pressure vessel contains seven to eight membrane elements depending on the treatment train configuration. The system operates at an average recovery rate of approximately 75%, with feed-water conductivity generally ranging between 2400 and 3300 µS/cm.

Unlike many laboratory-scale investigations, the present study is based on long-term industrial operating conditions where membrane performance is continuously affected by seasonal raw-water variability, hydraulic fluctuations, and fouling phenomena. Consequently, the system represents a relevant case study for evaluating the operational resilience of reverse osmosis desalination processes under realistic field conditions.

**Figure 1:**
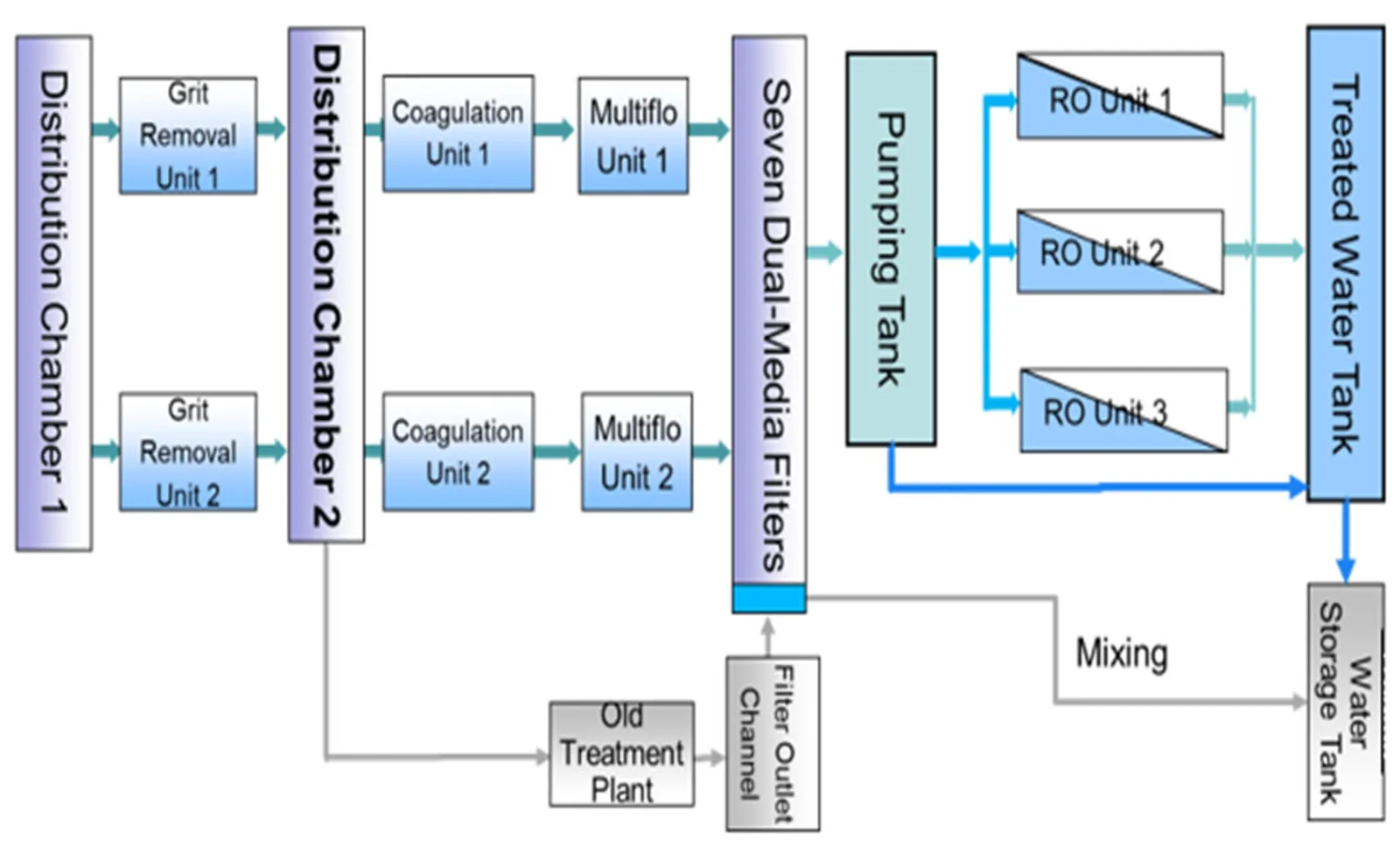
Diagram of the treatment process (coag-floc → settling → sand/multifiltration → MF → RO → chlorination). Arrows indicate the main direction of water flow; blue streams represent the treated-water line, while grey streams indicate mixing or side-flow connections.

The overall treatment configuration is presented in Figure 1, showing the sequence from grit removal and coagulation to multifiltration, pumping, RO units, and treated-water storage.

### 2.2. Data Collection, Monitoring, and Fouling Indicators

The study is based on monthly physicochemical analyses taken from various points on the reverse osmosis skids from 2020 to 2024: temperature, conductivity, total dissolved solids (TDS), hardness, pH, chlorides, permeate conductivity, differential pressure (ΔP), feed pressure, recovery rate, turbidity, SDI (Silt Density Index), chemical dosing conditions, and clean-in-place (CIP) operations. Such indicators are widely recognized as critical parameters for evaluating membrane fouling, hydraulic resistance, and process performance in industrial RO systems.

Sample collection was performed at multiple strategic locations along the treatment chain, including raw water, post-pretreatment water, reverse osmosis permeate, and concentrate streams.

Sampling campaigns were conducted on a monthly basis, and all principal physicochemical parameters were analyzed in triplicate in order to improve data reliability and reproducibility.

All analytical instruments were periodically calibrated according to manufacturer recommendations using certified reference solutions and standard calibration procedures.

To ensure data quality and analytical consistency, quality assurance and quality control (QA/QC) procedures were systematically implemented throughout the monitoring campaign. Calibration verification was performed before each analytical sequence, and measurement uncertainties were minimized through replicate analyses and routine instrument validation. Detection limits and instrumental precision were verified according to laboratory operational procedures. In addition, anomalous measurements and outliers were cross-checked against operational records and SCADA data prior to statistical interpretation.

Physicochemical analyses were carried out using calibrated laboratory and online analytical instruments according to standard methods for water and wastewater examination. Conductivity and pH measurements were performed using portable multiparameter probes equipped with automatic temperature compensation. Turbidity measurements were conducted using nephelometric methods, while ionic species were analyzed using standard titration, spectrophotometric, and ion-analysis techniques depending on the monitored parameter. All analytical instruments were periodically calibrated according to manufacturer recommendations using certified reference solutions and standard calibration procedures.

The hydraulic performance of the membranes was evaluated before and after CIP operations. For example, during a representative CIP sequence performed in 2023, the differential pressure of the first stage decreased from 2.9 bar to 1.7 bar after cleaning, while permeate conductivity improved from 106 µS/cm to 98 µS/cm.

The main operational parameters and fouling indicators used for long-term RO performance assessment are summarized in Table 1.

### 2.3. Water Quality Envelope and Treatment Objectives

The reverse osmosis (RO) desalination system investigated in this study was designed to operate under highly variable raw-water quality conditions typical of semi-arid environments exposed to seasonal hydrological fluctuations and progressive mineralization. Consequently, defining the inlet water quality envelope and the corresponding treatment objectives is essential for interpreting long-term membrane performance and operational stability.

During the monitoring period (2020–2024), the feed water exhibited relatively high mineralization levels, with conductivity values generally ranging between approximately 2400 and 3300 µS/cm and total dissolved solids (TDS) characteristic of brackish surface water conditions. The hydrochemical composition was dominated by calcium, magnesium, chloride, bicarbonate, and sulfate ions, which are recognized as major contributors to scaling and membrane fouling risks in reverse osmosis systems. In addition, turbidity and particulate loading exhibited seasonal variations associated with hydrological and climatic conditions.

To ensure stable membrane operation, the pretreatment train was designed to maintain low particulate fouling potential prior to RO treatment. Operational records indicated SDI values generally below 2 and turbidity values lower than 0.15 NTU before membrane filtration, confirming the effectiveness of the implemented pretreatment strategy. These values remain significantly below the commonly recommended SDI threshold of 5 for reverse osmosis membrane protection.

The principal treatment objective of the installation was the production of drinking water compliant with national and international quality requirements. The RO system was therefore operated to achieve high salt rejection performance and stable permeate quality under variable operating conditions. Throughout the monitoring period, permeate conductivity remained generally below 100 µS/cm, while salt rejection efficiency exceeded 90%, confirming the robustness of the desalination process despite feed-water variability.

The operational design basis of the plant also included scaling prevention and membrane-protection strategies through acid dosing, sodium metabisulfite injection for dechlorination, antiscalant addition, and cartridge microfiltration prior to reverse osmosis treatment. These operational measures played a critical role in limiting inorganic scaling, particulate accumulation, and biological fouling during long-term operation.

Overall, the definition of the water quality envelope and treatment objectives provides an essential framework for understanding the relationships among feed-water characteristics, pretreatment efficiency, membrane fouling behavior, and long-term operational stability in full-scale reverse osmosis systems.

### 2.4. Clean-in-Place (CIP) Strategy

Membrane cleaning operations were carried out according to an industrial Clean-in-Place (CIP) protocol combining alkaline cleaning, disinfection, and acidic cleaning stages. The alkaline cleaning stage was performed using solutions at concentrations of approximately 2%, with recirculation periods ranging from 4 to 8 h depending on fouling severity.

The cleaning protocol also included a dedicated disinfection step in order to mitigate biological contamination and biofilm development. Finally, acidic cleaning was applied to remove inorganic deposits and mineral scaling.

The implementation of combined alkaline and acidic cleaning strategies is consistent with international recommendations for controlling organic fouling, biofouling, and inorganic scaling in reverse osmosis systems. Recent studies have shown that optimized CIP protocols significantly improve membrane longevity and reduce irreversible fouling phenomena in long-term desalination operation.

### 2.5. Statistical Methodology

Statistical analyses were performed using Python 3.11 (Python Software Foundation, Wilmington, DE, USA) and scientific libraries including Pandas 2.2.2, NumPy 1.26.4, SciPy 1.13.1, Statsmodels 0.14.2, Scikit-learn 1.5.1, Matplotlib 3.9.0, Seaborn 0.13.2, and PyMannKendall 1.4.3. These tools were used to analyze the long-term operational and physicochemical dataset collected from the reverse osmosis desalination plant during the monitoring period extending from 2020 to 2024.

The adopted statistical framework was designed to evaluate hydrochemical variability, operational stability, temporal evolution, and the relationships between the principal physicochemical and operational parameters governing desalination performance under real industrial operating conditions.

Initially, descriptive statistical analyses were conducted for all monitored parameters, including the mean, median, minimum, maximum, standard deviation, quartiles, skewness, and coefficient of variation (CV). These statistical indicators enabled the characterization of the dispersion and temporal variability of the monitored variables, particularly conductivity, TDS, calcium, chloride, turbidity, SDI, temperature, alkalinity, and salt rejection efficiency.

To quantitatively assess process stability and operational consistency, the Operational Stability Index (OSI) was calculated according to the following equation:

OSI = 1 − CV(1)

where (CV) represents the coefficient of variation of the monitored parameter. Higher OSI values indicate lower variability and greater operational stability. This approach is consistent with previously recommended methodologies for the long-term evaluation of membrane-treatment systems and desalination-process performance.

Linear regression analyses were performed to investigate temporal trends and evaluate the relationships between hydrochemical variables and membrane-process indicators. Pearson correlation analyses were additionally conducted to identify the principal interactions between physicochemical and operational parameters. Correlation matrices and heatmaps were generated to improve the interpretation of multivariate relationships within the desalination system.

To strengthen the long-term temporal interpretation of the monitored parameters, advanced time-series analysis methods were incorporated into the statistical framework. Seasonal-Trend decomposition using Loess (STL) was applied to separate long-term trends, seasonal components, and residual variability for the principal monitored variables. This approach enabled the identification of progressive hydrochemical evolution and recurring seasonal fluctuations associated with environmental and operational conditions.

Stationarity analyses were conducted using the Augmented Dickey–Fuller (ADF) and Kwiatkowski–Phillips–Schmidt–Shin (KPSS) tests. These complementary tests were used to evaluate the temporal stability of the monitored series and determine whether the investigated variables exhibited stationary or non-stationary behavior over time.

Autocorrelation analyses were further performed using autocorrelation function (ACF) and partial autocorrelation function (PACF) plots to investigate temporal dependencies and persistence effects within the operational dataset.

Long-term monotonic trends were evaluated using the non-parametric Mann–Kendall trend test, while Sen’s slope estimator was used to quantify the magnitude and direction of the detected trends. These methods are particularly suitable for environmental and operational datasets characterized by non-normal distributions and seasonal variability.

To identify abrupt temporal changes and possible operational shifts, change-point analyses were conducted using Pettitt and Buishand homogeneity tests. These approaches enabled the detection of statistically significant discontinuities potentially associated with hydrological variability, pretreatment fluctuations, operational modifications, or membrane ageing effects.

Inter-annual comparisons were performed using both parametric and non-parametric statistical approaches depending on the distribution characteristics of the investigated variables. One-way analysis of variance (ANOVA) was applied for normally distributed datasets, while Kruskal–Wallis and Mann–Whitney tests were used for non-parametric comparisons. Prior to statistical testing, normality was verified using the Shapiro–Wilk test and homogeneity of variances using Levene’s test. Post-hoc analyses were performed using Tukey’s HSD test for parametric datasets and Dunn’s test for non-parametric analyses.

The monthly monitoring dataset consisted of approximately (n = 44) to (48) observations for most physicochemical and operational parameters. All statistical analyses were performed using a significance level of (*p* < 0.05).

Overall, the integration of descriptive statistics, multivariate analyses, time-series decomposition, stationarity testing, trend analyses, and change-point detection substantially improved the robustness of the long-term performance assessment and provided a more rigorous interpretation of membrane-process stability and desalination-system resilience under full-scale industrial operating conditions.

This statistical framework is consistent with the recommended approaches for the long-term evaluation of membrane treatment systems [4].

## 3. Results

### 3.1. Multi-Year Trends in Raw Water Quality (2020–2024)

**Figure 2:**
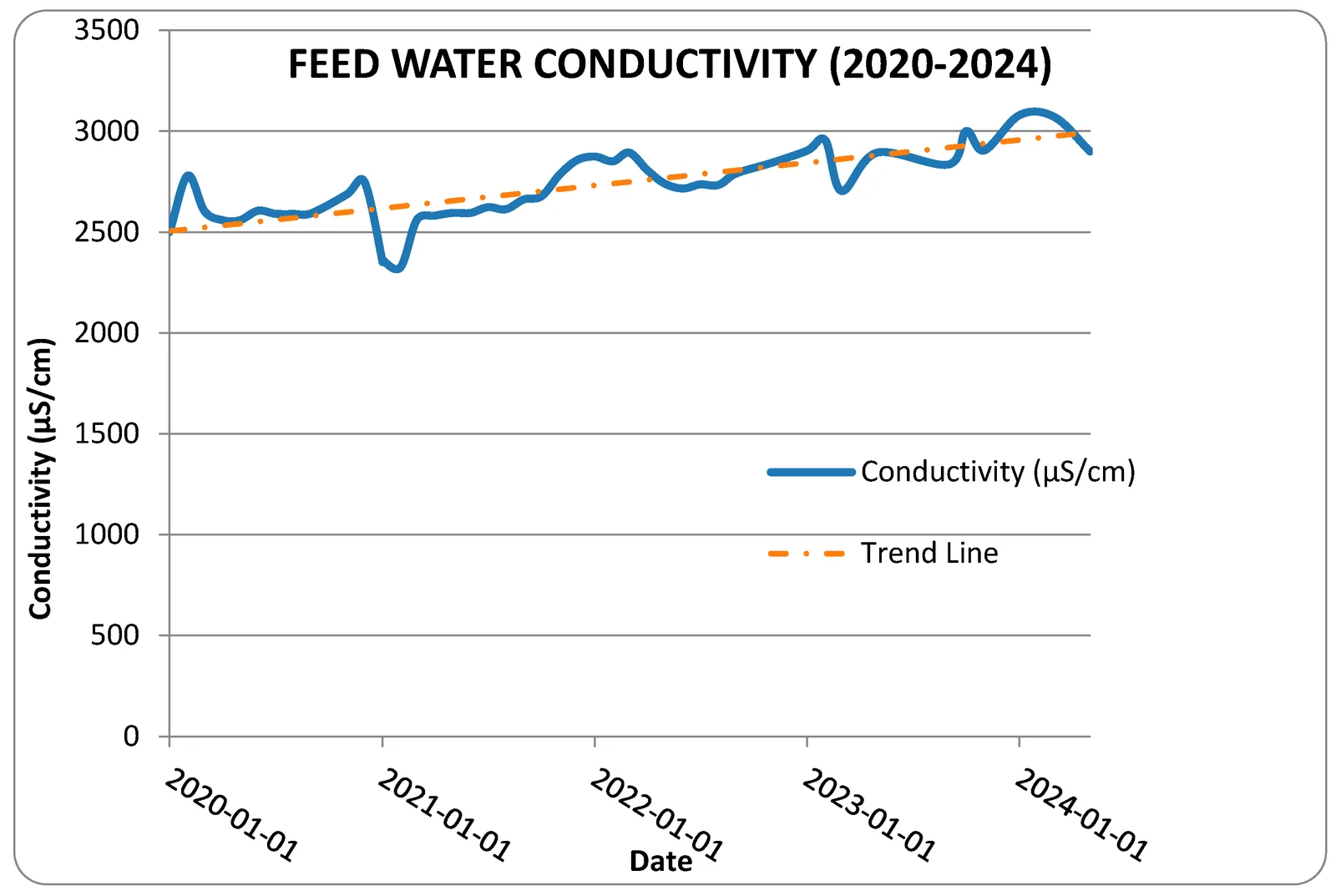
Multi-year trend in raw water quality (2020–2024).

**Figure 3:**
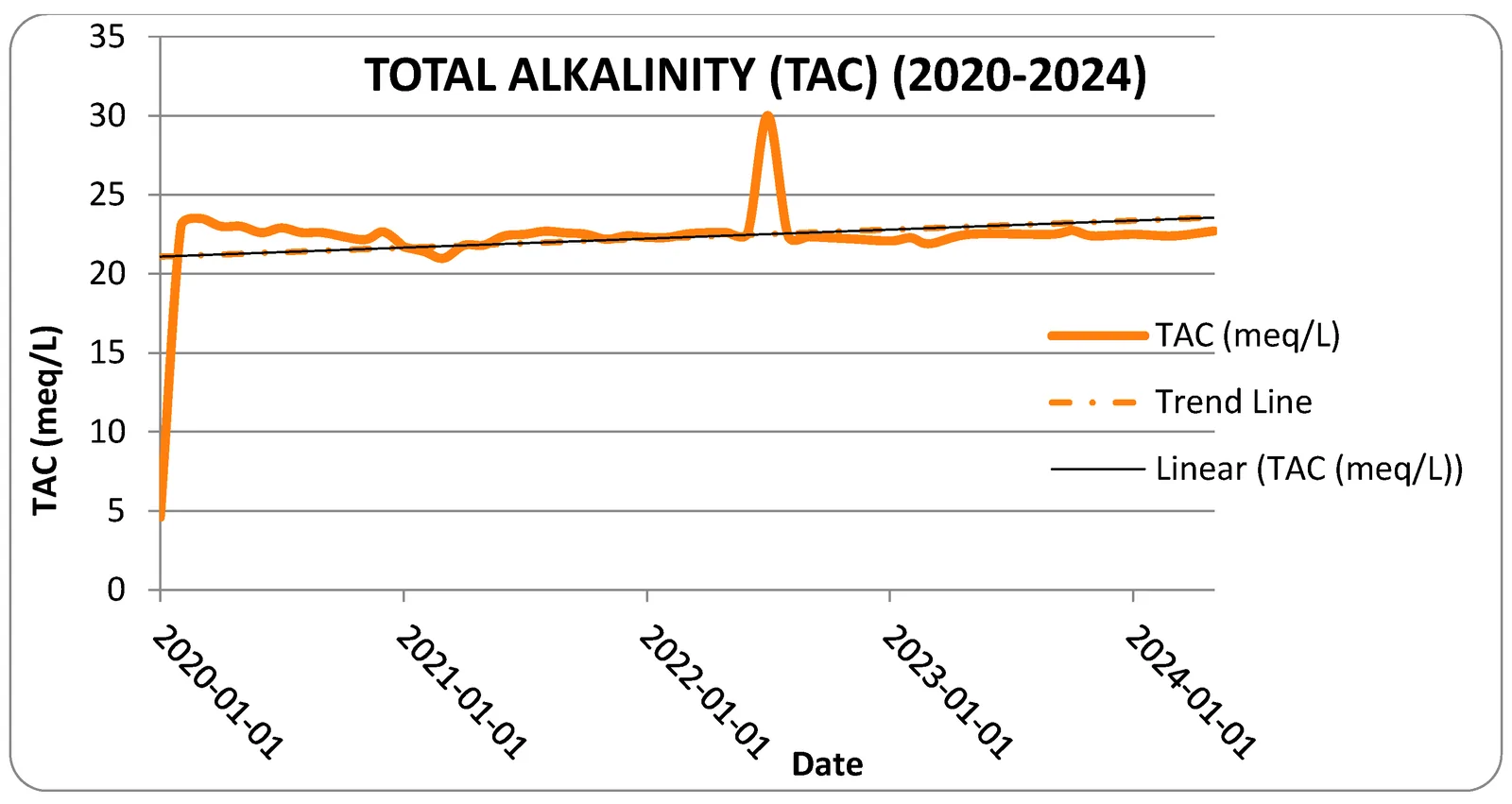
Temporal evolution of total alkalinity (2020–2024).

**Figure 4:**
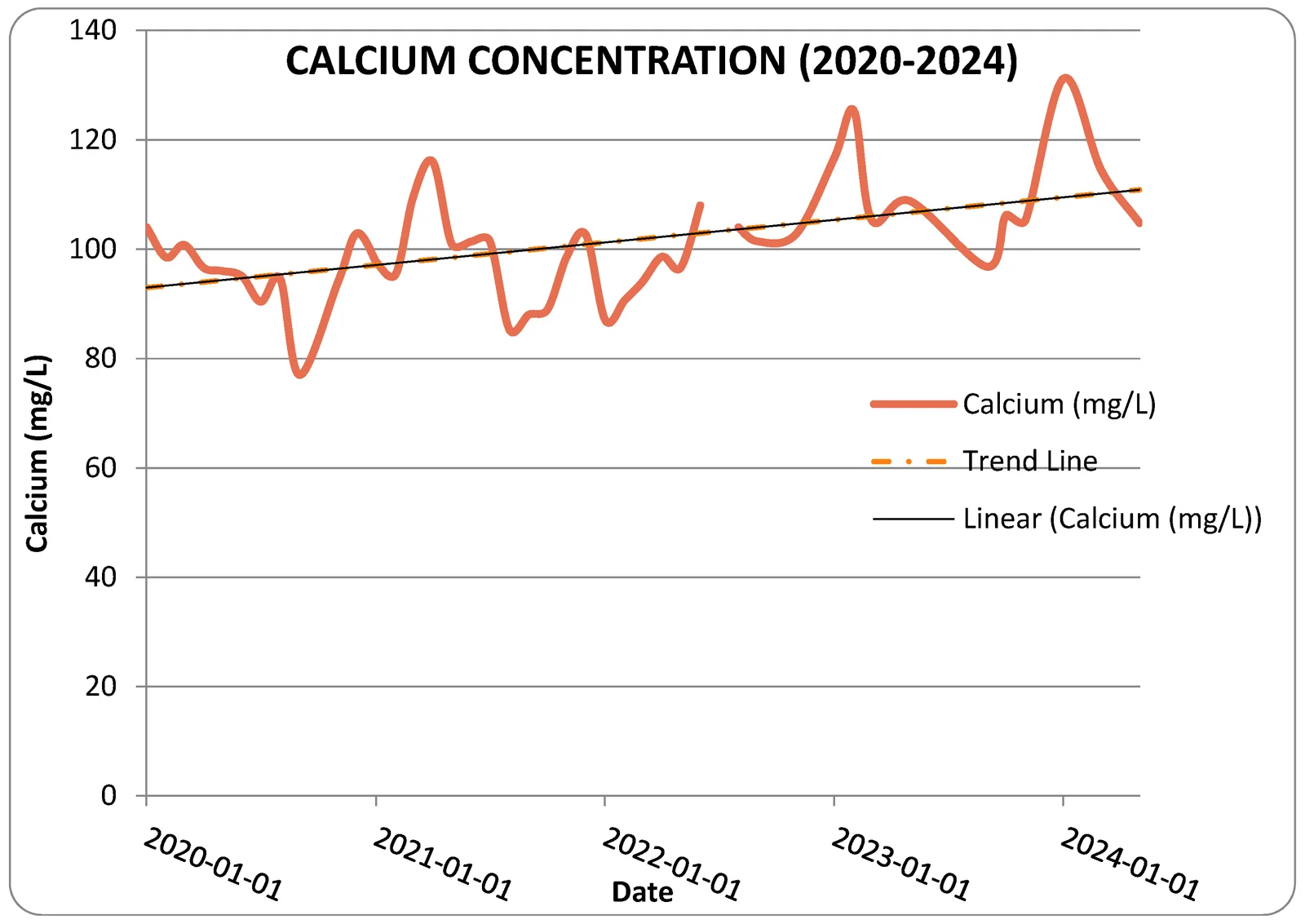
Temporal trend in calcium concentration (2020–2024).

**Figure 5:**
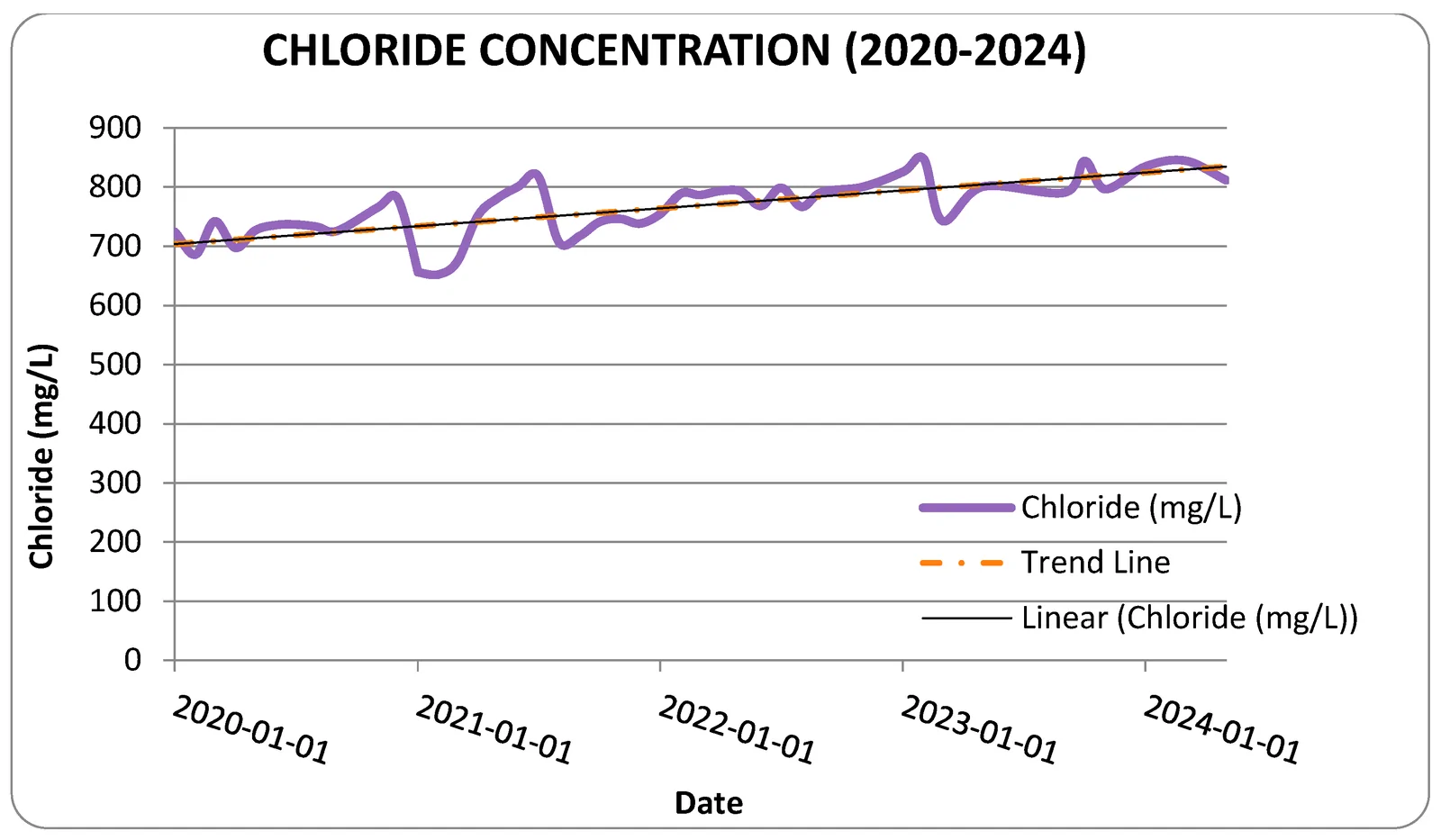
Temporal trend in chloride concentration (2020–2024).

**Figure 6:**
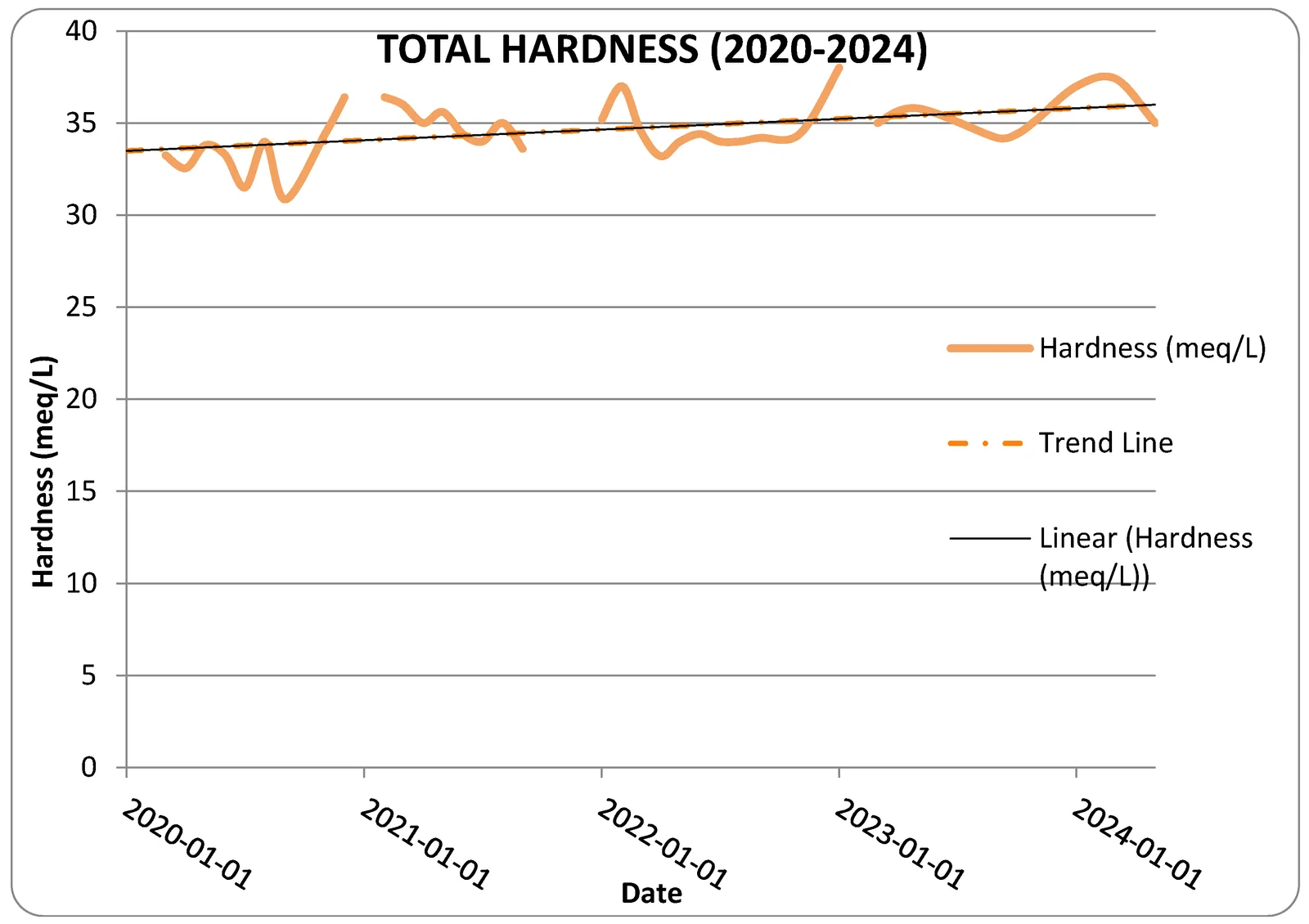
Time series of total hardness (2020–2024).

In fact, when we talk about salinity, we are indirectly referring to a set of parameters that directly or indirectly influence the physicochemical quality of water resources, especially in arid regions characterized by a hot climate, high temperatures, and low rainfall, which promote the evaporation of water resources and, consequently, a high concentration of mineral salts [5]. These areas are also characterized by significant agricultural potential, hence irrigation activities accompanied by significant anthropogenic pressures [6,7]. The corresponding temporal profiles are presented in Figure 2, Figure 3, Figure 4, Figure 5 and Figure 6, covering feed conductivity, total alkalinity, calcium, chloride, and total hardness, respectively.

The figure shows high and relatively stable total alkalinity throughout the monitoring period, reflecting a high buffering capacity mainly controlled by bicarbonate species. These conditions significantly increase the risk of calcium carbonate scaling in reverse osmosis systems.

Calcium concentrations remain high throughout the monitoring period, with moderate seasonal variability. The levels observed confirm that calcium is the main contributor to water hardness and a key factor in calcium carbonate scaling in reverse osmosis systems.

Chloride concentrations remain relatively high, reflecting the high mineralization of the raw water. As conservative tracers of salinity, chlorides are reliable indicators for assessing the overall ionic strength of the feed water and the efficiency of salt rejection by reverse osmosis membranes.

Total hardness falls mainly within the range from hard to very hard water. It is primarily controlled by the calcium concentration and alkalinity, confirming an inherently high potential for scaling, particularly under high-performance reverse osmosis operating conditions.

### 3.2. Buffering Effect of Conventional Pre-Treatment

**Figure 7:**
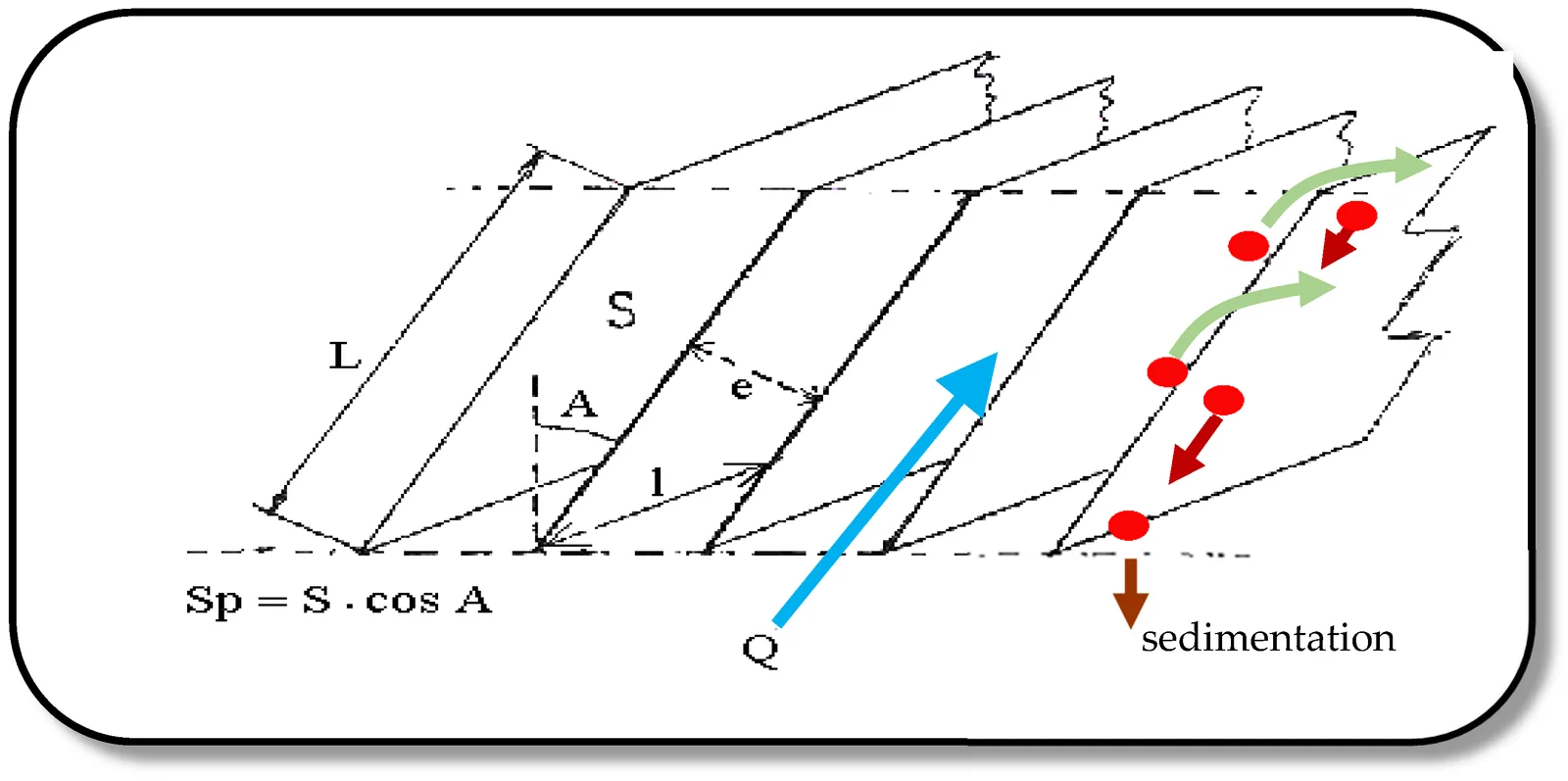
Turbidity reduction along the pre-treatment line. The arrows indicate the settling/flow direction, while the red circles represent suspended particles progressively removed during the settling stage.

Although the turbidity level of the raw water is sometimes as high as 20 NTU, the sequential pre-treatment steps such as coagulation-flocculation, sedimentation, and sand filtration guarantee a gradual and controlled decrease in suspended particles and lead to the ultimate turbidity level of less than 2 NTU before the microfiltration step. This particulate load stabilization is a prime cause of minimizing the risk of the downstream membrane fouling. Previous research confirms that the long-term stability and durability of reverse osmosis performance are strongly determined by the effectiveness of pre-treatment [8]. This removal mechanism is illustrated in Figure 7.

### 3.3. Stability and Structuring Role of Microfiltration

**Figure 8:**
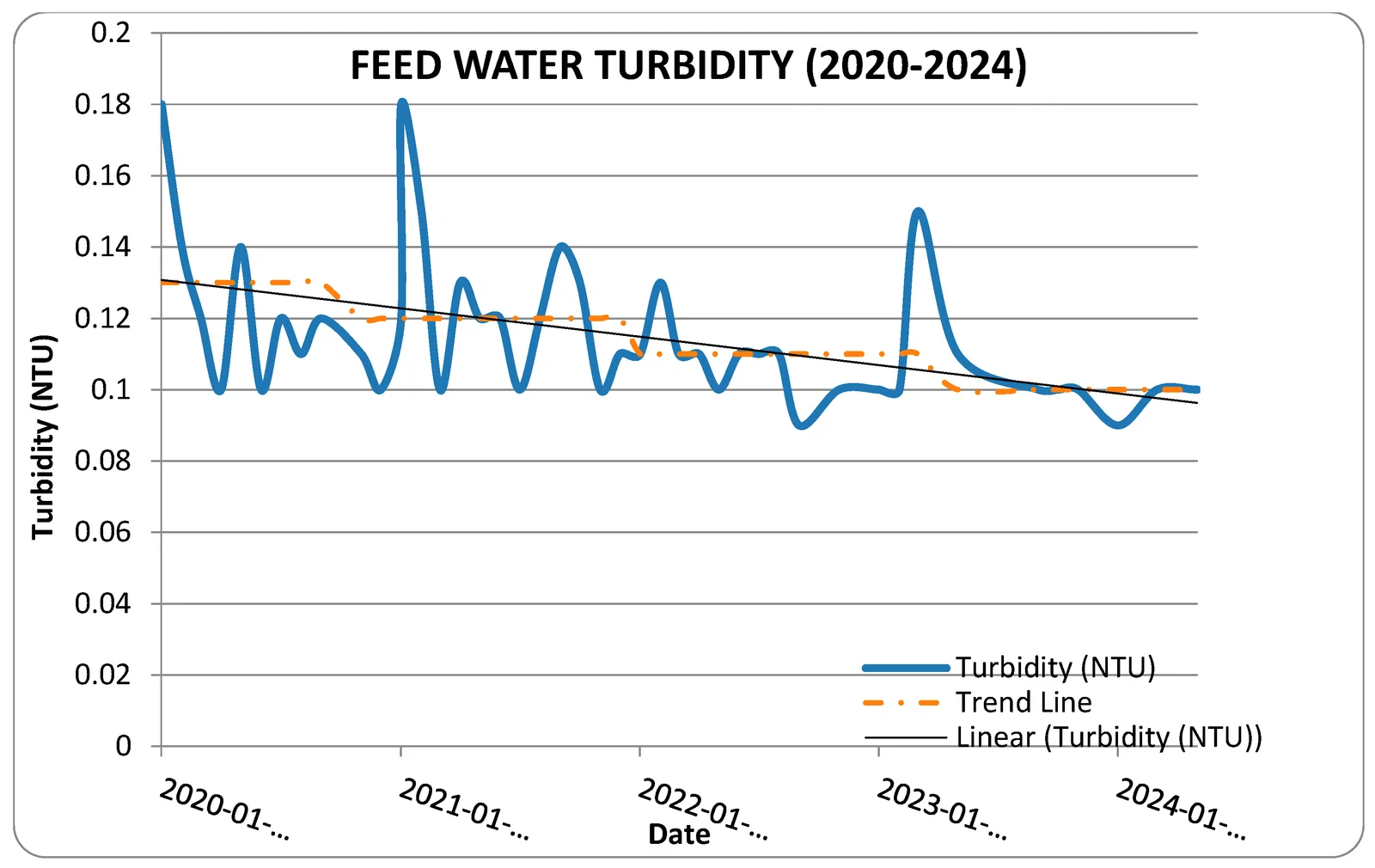
Box plot of turbidity after microfiltration.

As shown in Figure 8, the very low variation observed after the microfiltration stage, with median turbidity values below 0.15 NTU and low coefficients of variation, indicates that the pre-treatment system can remain operational over a long period. Microfiltration is a powerful physical barrier in eliminating fine particles and colloidal matter and, thus, a significant reduction in the particle load to the reverse osmosis units. This feed water stabilization is also important in reducing the mechanisms of membrane fouling, especially the particulate and organic fouling that form part of the mechanisms that influence the long-term operation of reverse osmosis systems. The introduction of microfiltration into the pre-treatment chain will, therefore, enhance the reliability and longevity of the membrane process. These findings are in line with earlier research pointing out the necessity of membrane pre-treatment to ensure stable reverse osmosis operation [9,10,11].

### 3.4. Performance and Resilience of the Reverse Osmosis System

The multi-year comparison between feed-water and permeate conductivity confirms the resilience of the reverse osmosis process under variable raw-water quality. Although feed-water conductivity showed progressive variability associated with hydrochemical evolution, permeate conductivity remained comparatively stable throughout the monitoring period and salt rejection was consistently maintained above 90%. These results indicate that the observed increase in raw-water mineralization was effectively buffered by the pretreatment and reverse osmosis treatment chain, confirming the operational robustness of the full-scale system.

### 3.5. Multivariate Analysis and Correlations Between Key Parameters

**Figure 9:**
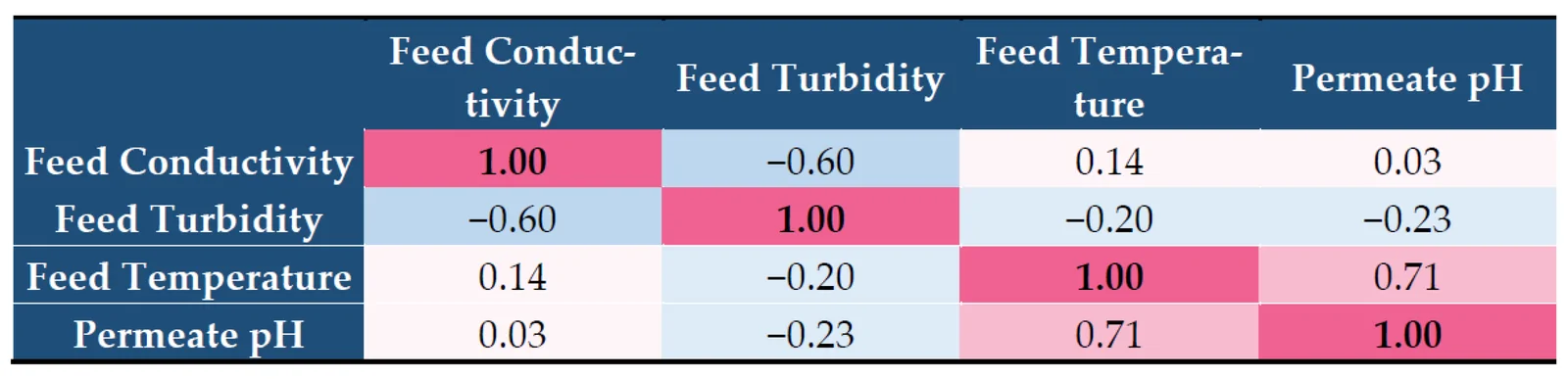
Revised correlation heatmap for operational and physicochemical indicators (2020–2024). Pink shading highlights stronger positive correlations, blue shading indicates negative correlations, dark-blue cells correspond to variable labels, and bold values indicate diagonal self-correlations.

As shown in Figure 9, the correlation matrix derived from the physico-chemical parameters monitored between 2020 and 2024 shows a strong positive association between temperature and pH (r = 0.71), reflecting the effect of thermal conditions on the chemical equilibria of raw water. In contrast, conductivity shows only a weak positive correlation with temperature (r = 0.14), suggesting that warmer conditions may promote mineralization to a limited extent without, however, being the determining factor. The near absence of a correlation between pH and conductivity (r = 0.03) further indicates that the system retains a relatively stable buffering capacity despite the gradual increase in the ion content.

Overall, these results support the interpretation that the gradual deterioration of raw water quality is primarily driven by hydrological and climatic constraints rather than by intrinsic chemical instability. Furthermore, the absence of a significant correlation between the turbidity of the pre- and post-microfiltration turbidity and the conductivity of the permeate confirms that particulate fouling has remained effectively mitigated, in line with the classical theory of membrane fouling [12].

**Figure 10:**
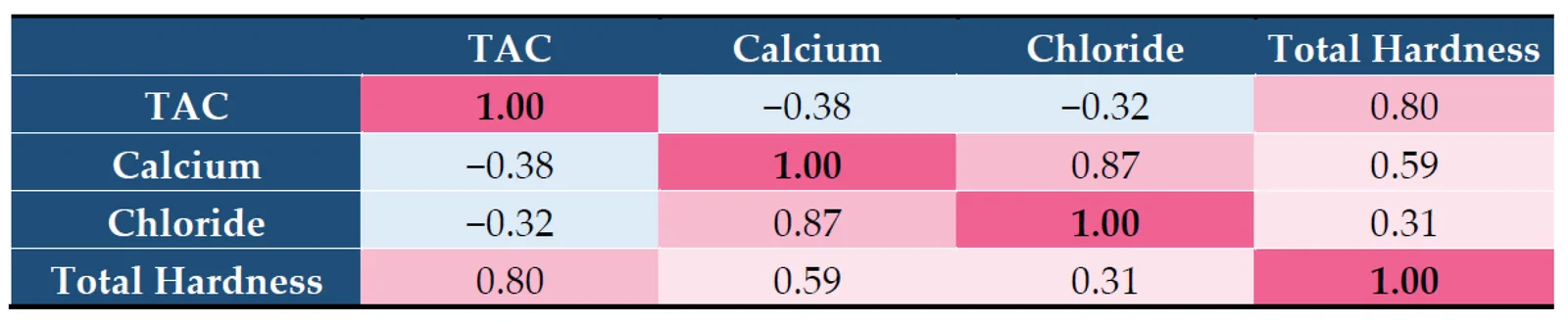
Revised Pearson correlation matrix for the main mineralization parameters (2020–2024). Pink shading highlights stronger positive correlations, blue shading indicates negative correlations, dark-blue cells correspond to variable labels, and bold values indicate diagonal self-correlations.

The clarified correlation matrices and consistent color scale improve the interpretation of positive and negative relationships and support the conclusion that feed-water mineralization is mainly controlled by hydrochemical evolution, while RO permeate quality remains operationally stable. The detailed mineralization relationships are presented in Figure 10.

Pearson correlation analysis showed a high positive relationship between the calcium concentration and chloride concentration (r = 0.87), which suggests that these two variables can share the same origin of mineralization or are related to some joint processes responsible for raising salinity levels. Total alkalinity also exhibited a strong correlation with total hardness (r = 0.80) due to the role played by carbonates in relation to calcium and magnesium. Meanwhile, the presence of weak inverse relationships between alkalinity and calcium (r = −0.38), as well as between alkalinity and chlorides (r = −0.32), implies different geochemical processes affecting these variables. Such correlations have been described previously in water mineralized prior to being treated in reverse osmosis facilities [13,14,15]. The signs and magnitudes of the reported correlation coefficients were checked and confirmed against the recalculated matrix.

**Figure 11:**
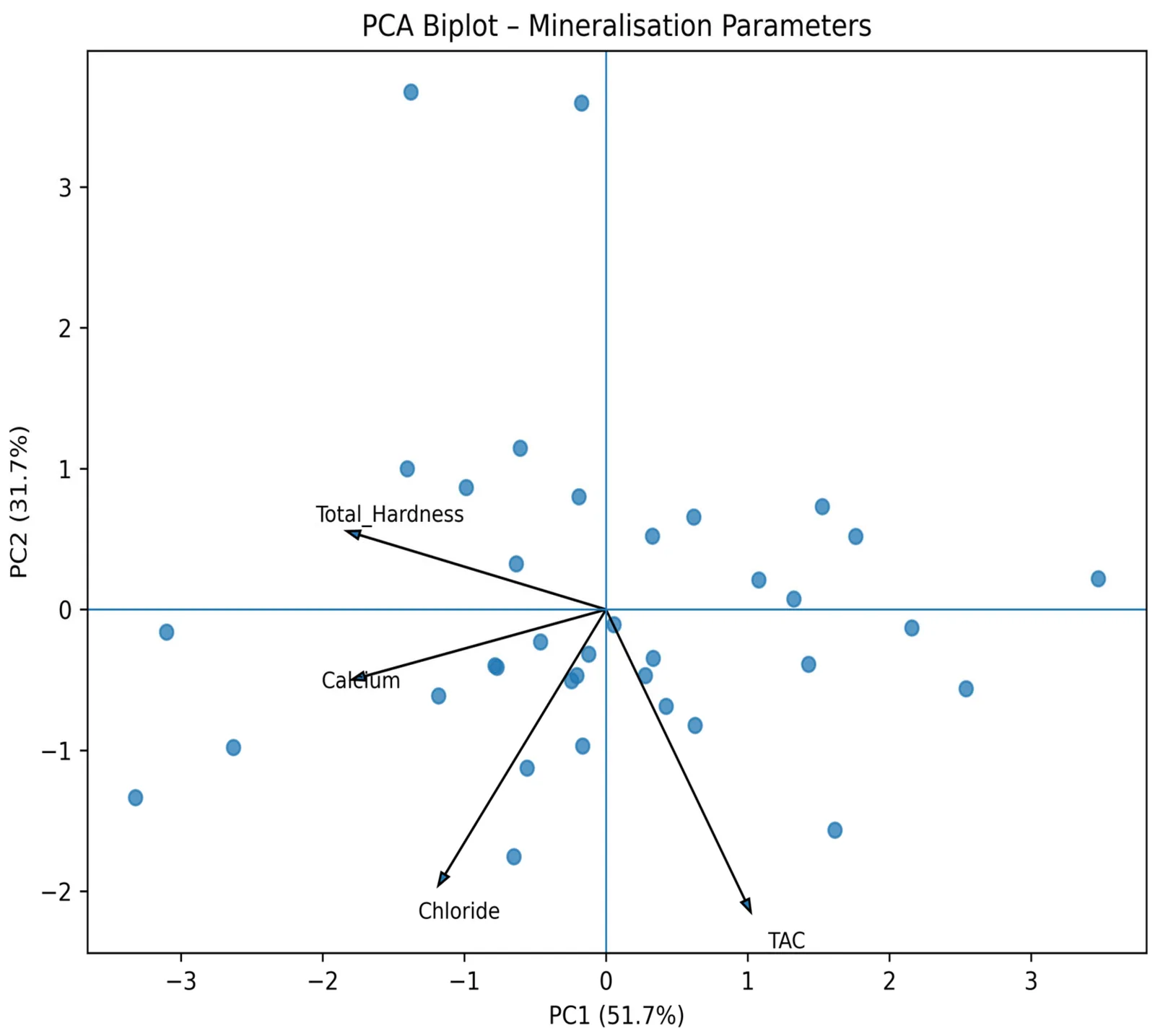
Principal component analysis (PCA) biplot illustrating the relationships between mineralization parameters. Dots represent monthly observations, while arrows represent the direction and strength of variable loadings.

As illustrated in Figure 11, the biplot obtained from the PCA analysis indicates that there are two primary hydrochemical controls. PC1, mostly influenced by the total alkalinity and hardness, represents the role of carbonate chemistry and the scaling potential. On the other hand, PC2, mostly influenced by the chloride content, represents the salinity control, which seems to be independent of carbonate chemistry. Such separation shows that the two parameters, salinity and the potential for scaling, are influenced by different hydrochemical mechanisms, a finding supported by previous literature on membrane water treatment plants [15,16,17,18].

**Figure 12:**
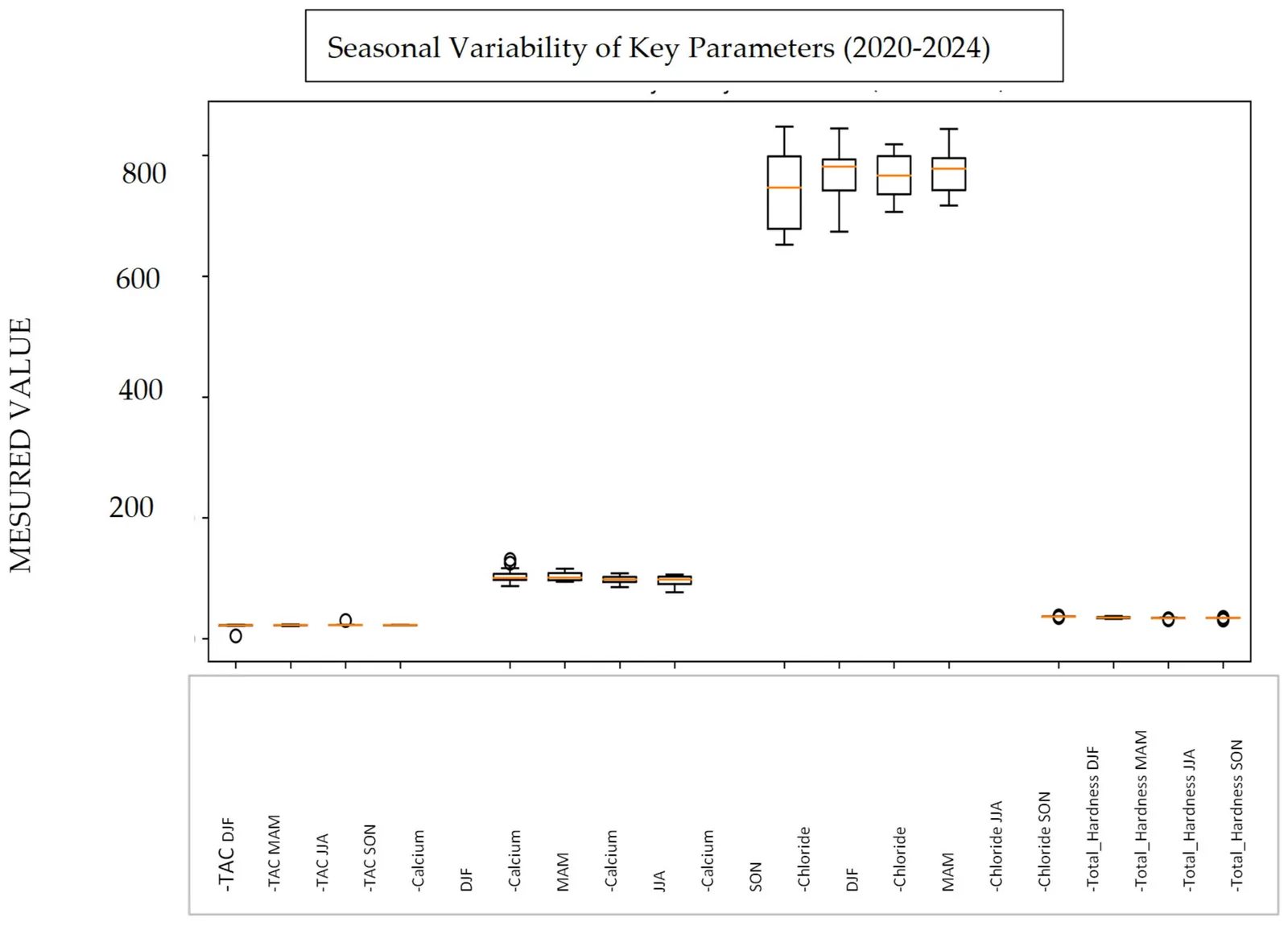
Seasonal variability in key parameters (2020–2024).

As shown in Figure 12, seasonal boxplots illustrate moderate but consistent variations on an annual basis with regard to alkalinities, calcium, chlorides, and total hardness. The greater median concentrations found during the dry seasons (MAM, SON) demonstrate the effect of the increased concentration due to low diluting capability and evaporation rates, whereas smaller medians obtained during wet seasons (DJF, JJA) are the product of hydrological dilution. In spite of the differences in concentrations on an annual basis, the narrowness of interquartile ranges indicates the stability of the mineralization process, which is common for hydrologically controlled flows and groundwater-based processes [13,19,20].

**Figure 13:**
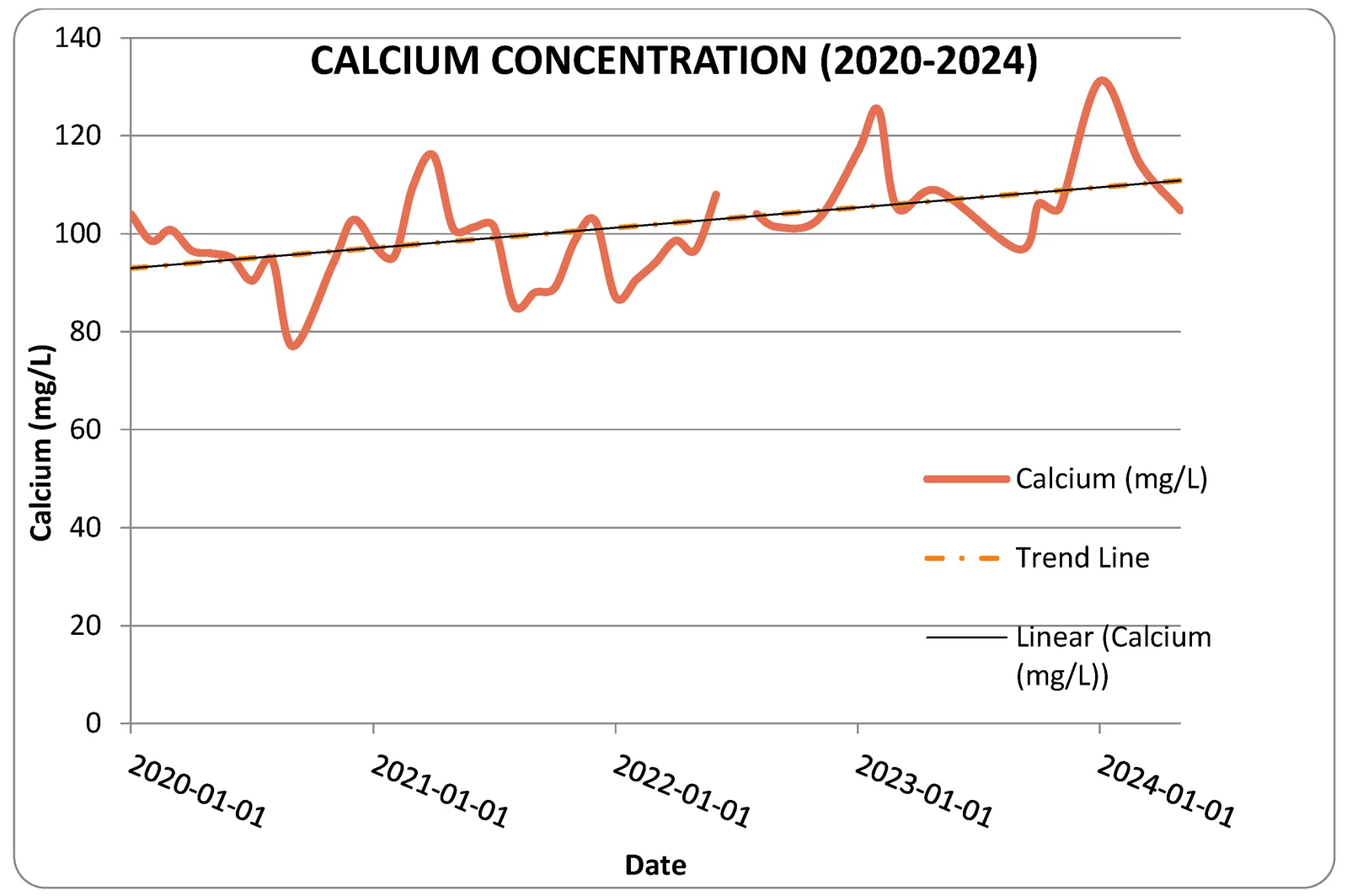
Linear regression of calcium concentration showing the 95% confidence interval.

As shown in Figure 13, the regression analysis for calcium shows an increase in its concentration during the observation period, although not statistically significant at the 95% level of confidence. Therefore, calcium fluctuations should be attributed to short-term seasonal or hydrological changes, rather than the long-term degradation of raw water quality. It is worth mentioning that including the 95% confidence interval makes it possible to properly estimate the regression uncertainty according to good practices in environmental time-series analysis [15,16,21].

The isolated outlier observed during mid-2022 corresponds to a temporary operational disturbance and maintenance intervention rather than a persistent hydrochemical transition.

**Figure 14:**
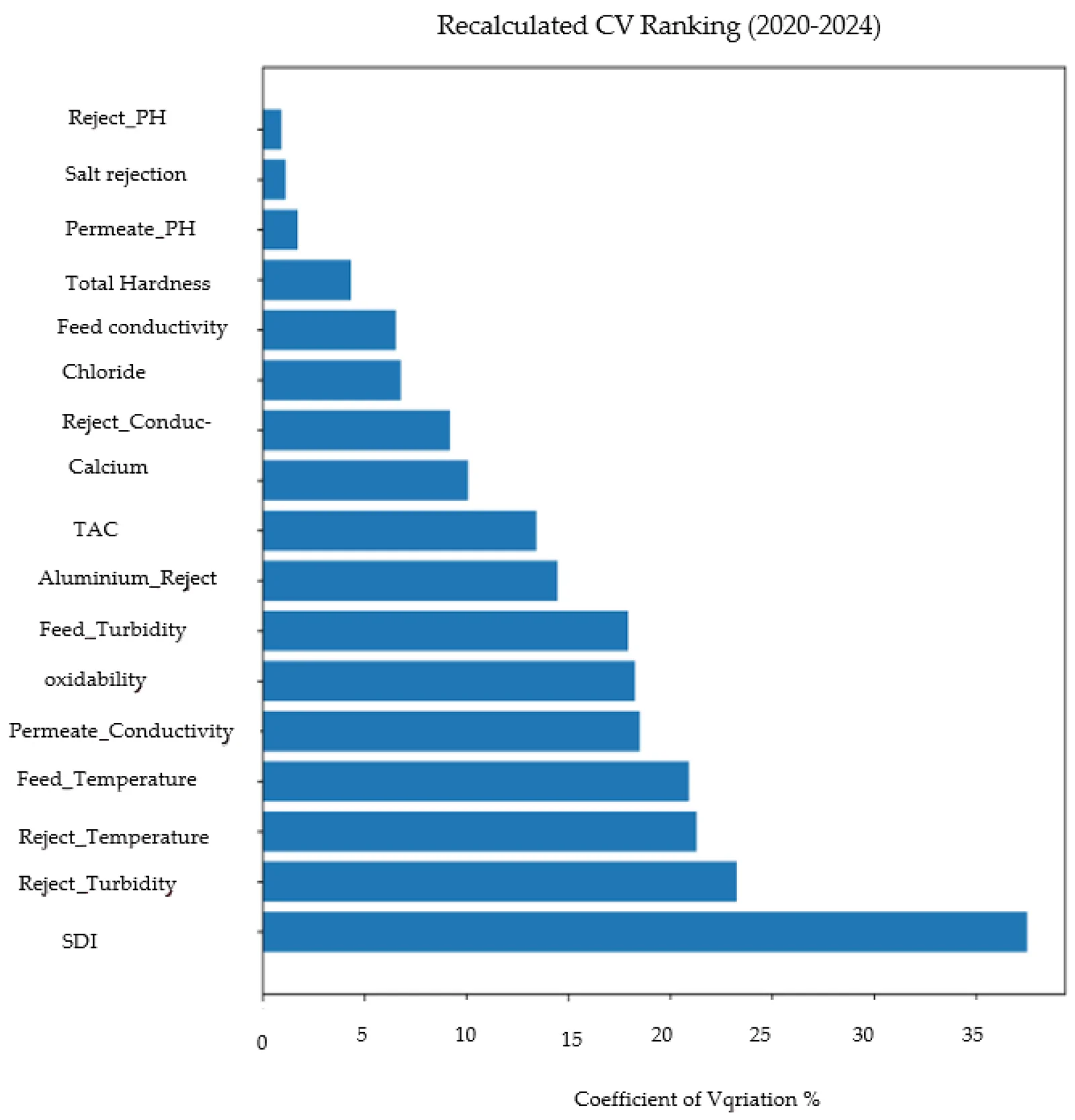
Ranking of the variability of monitored parameters based on the coefficient of variation (2020–2024).

As shown in Figure 14, coefficient of variation analysis indicates a clear differentiation between parameters’ time stability. The parameter of calcium demonstrates the highest coefficient of relative variation and implies a higher sensitivity to hydrological and other factors associated with changes throughout different seasons. In turn, the value of total alkalinity demonstrates the lowest variability, which indicates the presence of a significant buffer capacity of the system. It becomes especially important to consider these values when operating the reverse osmosis system since parameters demonstrating high variability need special monitoring in order to avoid the scaling of membranes and other negative effects [13,17,22]. The stationarity results, trend statistics, change-point detection, and descriptive statistics are summarized in Table 2, Table 3, Table 4 and Table 5, respectively.

**Figure 15:**
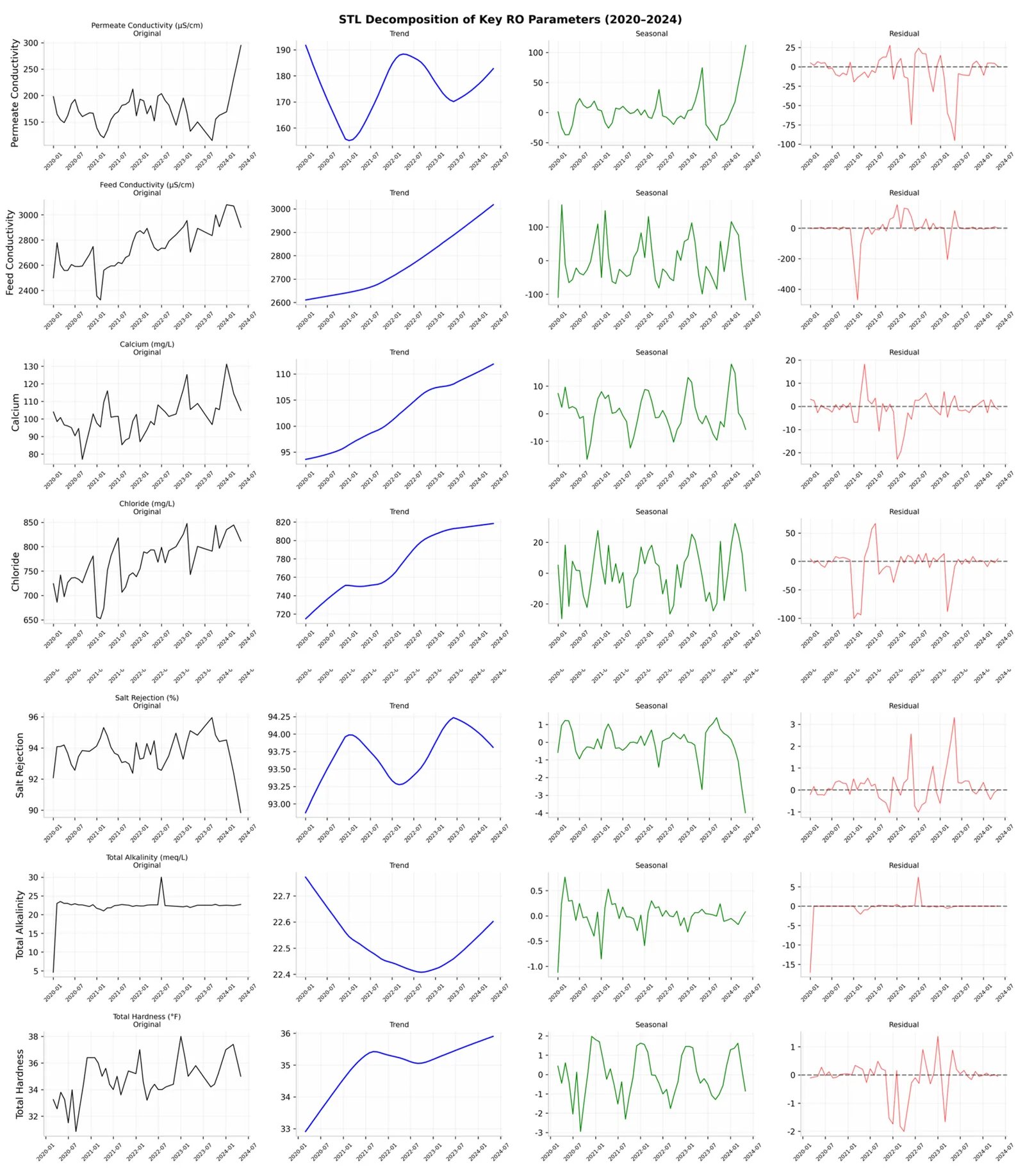
Enlarged STL decomposition of key RO parameters (2020–2024), presented in two panels for improved readability. Dashed lines indicate trend components, and the colors distinguish the decomposed time-series components.

As shown in Figure 15, the time series analysis was substantially strengthened by incorporating rigorous statistical methods. Stationarity testing (ADF and KPSS) confirmed that feed water conductivity and chloride are non-stationary (*p* < 0.05), indicating persistent trends in raw water mineralization. In contrast, salt rejection, total alkalinity, and total hardness were confirmed to be stationary, demonstrating robust operational control. The Mann-Kendall trend testing revealed statistically significant increasing trends in feed conductivity (+128.0 µS/cm/year, *p* < 0.001), calcium (+3.77 mg/L/year, *p* = 0.002), chloride (+33.9 mg/L/year, *p* < 0.001), and total hardness (+0.72 °F/year, *p* = 0.009). Notably, permeate conductivity and salt rejection showed no significant monotonic trends (*p* > 0.05), confirming the resilience of the RO system despite upstream degradation. Change point detection (Pettitt and Buishand U tests) converged on October 2021 for feed conductivity, January 2022 for chloride, and May 2022 for calcium, marking a critical transition period coinciding with documented regional hydrological stress [23,24]. Autocorrelation analysis revealed strong temporal persistence in feed parameters (ACF significant up to 6–8 lags) but weak autocorrelation in performance indicators, consistent with stable, memory-less process behavior. These statistical outputs also provide a basis for long-term stability assessment and future predictive fouling-monitoring approaches [25,26,27].

The observed trend in raw water quality parameters reflects the natural hydrochemical variability associated with seasonal climatic patterns and inter-annual hydrological fluctuations in the semi-arid region, rather than an indication of irreversible water quality deterioration. The stability of treated water quality despite this variability demonstrates the robustness of the implemented treatment strategy.

### 3.6. Membrane Fouling Diagnosis and Autopsy Analysis

**Figure 16:**
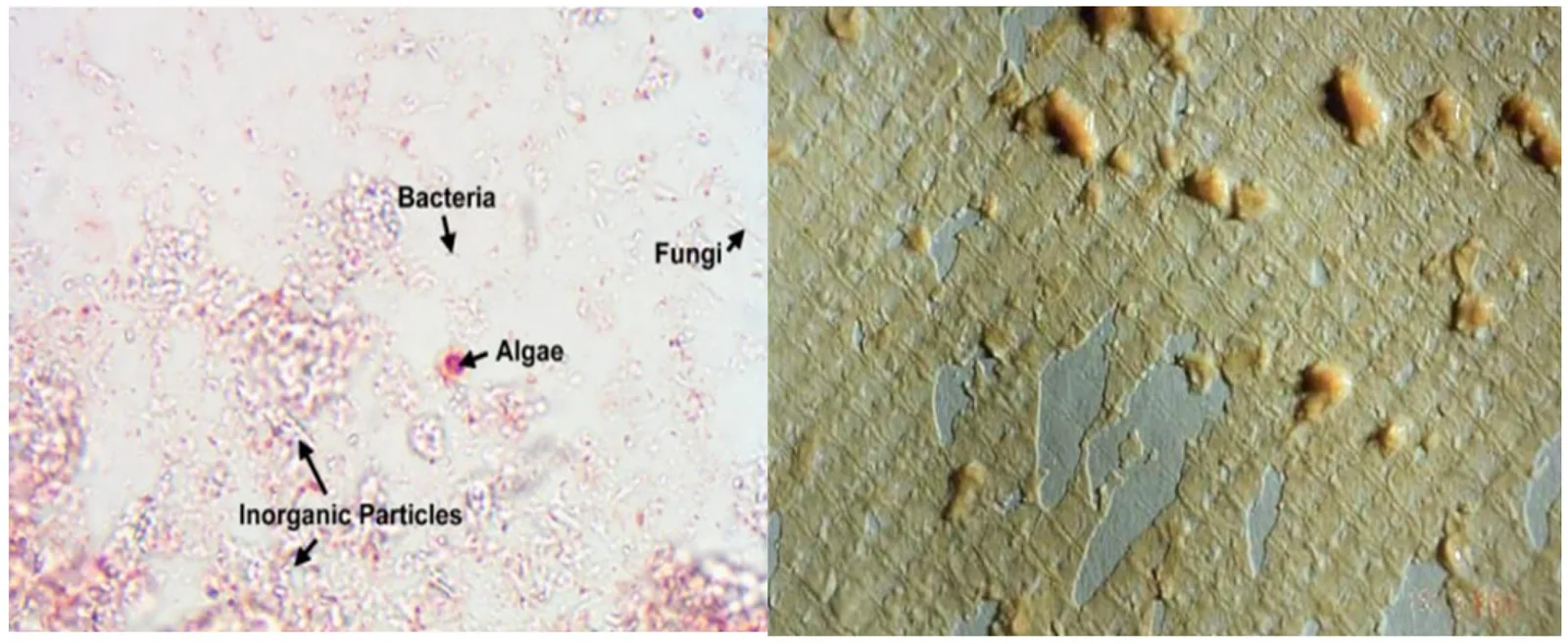
Biofilm at the surface of a membrane.

To further investigate the mechanisms affecting long-term membrane performance, an autopsy analysis was conducted on several spiral-wound membrane elements removed from the industrial installation. The autopsy included non-destructive testing, visual inspection, ATP activity measurements, FTIR analysis, CEI analysis, and microscopic characterization. The representative biofilm morphology observed during the autopsy is shown in Figure 16.

The analysis revealed the coexistence of inorganic scaling, biological fouling, and oxidative degradation phenomena. Significant biological activity was detected on several membrane surfaces, with ATP measurements indicating highly active biofouling and the presence of bacteria, fungi, algae and bioslime structures. In addition, FTIR and SEM/EDX analyses identified silica-based deposits and clay particles on the membrane surface, confirming the contribution of inorganic scaling mechanisms.

One membrane element also exhibited evidence of chlorine-induced degradation, indicating incomplete dechlorination upstream of the reverse osmosis system. This observation highlights the importance of sodium metabisulfite dosing and residual chlorine monitoring in protecting polyamide membranes against oxidative damage. Similar degradation mechanisms have been widely reported in the membrane desalination literature.

The autopsy investigation therefore confirms that long-term membrane performance is governed not only by feed-water salinity but also by the combined effects of biofouling, particulate accumulation, silica scaling, and operational conditions. These findings reinforce the importance of integrated pretreatment and proactive monitoring strategies for ensuring sustainable reverse osmosis operation under highly mineralized water conditions.

## 4. Integrated Scientific Discussion

### 4.1. Hydrochemical Analyses of Raw Water Mineralization

Hydrochemical features of the raw water found in Khénifra are typical of the geochemical indicators typically found in semi-arid environments, in which mineralization is highly dependent on climatic and geological conditions. The reduced recharge rates, high evaporation, and increased residence time of groundwater in the carbonate-rich geological formations are frequently associated with the progressive concentration of dissolved ions in such environments. These circumstances favor the solution of carbonate minerals, especially calcite and dolomite, that result in large amounts of calcium, bicarbonates, and overall hardness of the aqueous matrix. The high correlation among the variables, alkalinity, calcium, and total hardness, reinforces the hypothesis that the carbonate dissolution is the prevailing hydrochemical process that controls the mineralization in the system under investigation. In carbonate water bodies, the balance among dissolved carbon dioxide, ions of bicarbonate, and carbonate minerals is important to balance the water chemistry. These equilibria can change with variations in temperature, hydrological recharge, and the groundwater residence time, resulting in seasonal changes in the levels of alkalinity and hardness. This hydrochemical behavior is common in highly mineralized waters that are used in the production of drinking water and in desalination. It was previously demonstrated that carbonate-dominated mineralization profiles are especially crucial to membrane-treatment systems, as they directly affect the potential of scaling and the stability of operations. In these circumstances, precipitation of calcium carbonate is one of the most typical fouling mechanisms of reverse osmosis membranes.

### 4.2. Implications for Scaling Mechanisms in Reverse Osmosis Systems

The high content of carbonate chemistry in the raw water composition has a telling effect on the fouling behavior of reverse osmosis systems. Calcium carbonate scaling is known to present one of the most critical operational issues of the membrane desalination processes. The saturation state of the solution is mainly determined by the precipitation of CaCO_3_ and, thus, is influenced by various factors, such as the calcium concentration, alkalinity, pH, temperature, and ionic strength. One of the most widespread indicators of scaling propensity in water treatment system is the Langelier Saturation Index (LSI). A positive LSI value implies that there is calcium carbonate supersaturation meaning that the conditions are favorable for precipitation. However, with reverse osmosis systems, the scaling risk is usually reduced when the LSI is computed purely based on the chemistry of the bulk feed water. This is a limitation based on the fact that physicochemical conditions at the membrane surface can vary widely with the bulk solution. When operating in reverse osmosis, there is the effect of concentration polarization, which refers to the accumulation of rejected ions around the membrane interface, increasing the ionic strength and supersaturation level locally. Moreover, the ionic concentration in the concentrate stream is also increased by the recovery rate used in the membrane modules. This may result in the effective LSI at the membrane surface being much greater than that calculated by bulk water. A number of studies have noted the need to consider such localized effects in scaling risks in membrane systems. Otherwise, it can result in a low estimation of the scaling potential and insufficient strategies of operational control.

### 4.3. Role of Pretreatment in Reducing Fouling and Scaling

The findings of this research demonstrate the critical role of the pretreatment chain in ensuring stable reverse osmosis operation at high levels of mineralization. Coagulation–flocculation, sedimentation, sand filtration, and microfiltration combine to offer a multi-barrier treatment that greatly decreases the particulate and colloidal loads prior to the feed water entering the reverse osmosis units. More specifically, microfiltration is a very useful physical barrier, which guarantees a steady quality of feed water to the membrane modules. The extremely low turbidity values following microfiltration attest to the fact that the particle load by which the reverse osmosis system is fed is kept low. This stabilization in the feed water greatly minimizes the chances of particles fouling it and thus leading to faster membrane degradation and higher operating expenses. In addition to particle removal, effective pre-treatment also indirectly helps to mitigate scaling. Pre-treatment operations eliminate suspended solids and colloids which may serve as nucleation sites in mineral precipitation and thus minimize the possibility of heterogeneous scaling processes occurring inside the membrane modules.

### 4.4. Operational Resilience of the Reverse Osmosis System

Despite the gradual increase in raw water mineralization observed during the monitoring period, the reverse osmosis system maintained remarkably stable performance. Consistent permeate conductivity levels and sustained salt rejection rates exceeding 90% indicate that the system is operating under robust conditions.

This resilience reflects the combined effects of appropriate system design, effective pretreatment, and optimized operational management. The ability of the reverse osmosis process to maintain stable permeate quality despite fluctuations in the chemical composition of the feed water demonstrates the intrinsic robustness of well-designed membrane desalination systems.

From an operational perspective, maintaining this stability requires the continuous monitoring of key process parameters. The recovery rate, transmembrane pressure, and antiscalant dosage must be carefully adjusted to account for variations in feed water quality. Failure to maintain such control can lead to a gradual deterioration in performance due to scaling or fouling processes.

### 4.5. Operational Monitoring and Data-Driven Management

The long-term monitoring data that is analyzed in the present study offer useful information about the dynamics of the functioning of the Khénifra desalination plant. The constant data gathering and statistical analysis will help the plant operators to identify emerging trends, compare the performance of the treatment, and predict the possible operational risks. Over the past few years, the collaboration of digital monitoring and sophisticated data analytics have gained more significance in the management of water treatment facilities. In this study, operational dashboards are tools that allow visualizing key performance indicators and can be used to make evidence-based decisions. The fact that the statistical analysis methods are applied to the operational datasets also contributes to the identification of correlations and underlying mechanisms that cannot be easily noticed in the course of routine monitoring. Indicatively, the correlation and PCA analyses that were conducted in this study gave important information about the hydrochemical mechanisms that control the mineralization of raw water.

### 4.6. Towards Predictive Operations and Digital Twins

Another finding of this study is the possibility of introducing sophisticated modelling and predictive analytics into the operation of reverse osmosis systems. The increased access to operational datasets provides new opportunities to create digital tools that can facilitate predictive maintenance and process optimization. One of the most promising ways in this area is Digital Twin technologies. A Digital Twin is a virtual model of a physical system that combines real-time operational data with predictive models to model the behavior of the system in various operating conditions. A combination of statistical monitoring, machine learning algorithms, and process simulation models can be used to generate useful decision-support tools to plant operators through Digital Twins. These systems can be applied to predict scaling risks, optimize chemical dosing strategies, and improve energy efficiency. It is thus possible that the development of predictive models into operational monitoring systems would greatly enhance the long-term sustainability and reliability of membrane desalination plants. Limitations and future research directions are presented.

### 4.7. Limitations and Future Research Directions

Although this study offered some valuable information, a number of limitations should be noted. To begin with, the analysis was based on physico-chemical parameters that were mainly recorded in operational dashboards. Other operation indicators like the sediment density index (SDI) were not found in continuous forms like the variation in pressure drop across the modules of membranes and specific energy consumption. Such parameters might be used to obtain important supplementary data about the membrane fouling dynamics and the system performance. The need to include these indicators in future studies would enable a more thorough evaluation of reverse osmosis system performance. Moreover, more predictive opportunities may be provided by using more sophisticated data-driven strategies, especially machine learning models. This would enable the creation of early warning systems that are able to predict scaling or fouling events and prevent them, before they can occur and impact system performance.

The operational dataset generated during this long-term monitoring study may support the future development of predictive maintenance and Digital Twin frameworks for reverse osmosis supervision and optimization.

## 5. Conclusions

The sophisticated statistical analysis of the operational data during the 2020–2024 monitoring period shows that the Khénifra demineralization plant has stable operational stability and treatment efficiency, even though the quality of raw water has gradually declined. Specifically, the microfiltration-reverse osmosis treatment chain has already been demonstrated to be extremely resilient to seasonal and interannual fluctuations.

The results clearly show that, despite a progressive increase in feed-water mineralization, the treatment chain maintained robust desalination performance, stable permeate quality, and salt rejection consistently above 90%. The improved presentation of the statistical outputs, especially the correlation matrices, further strengthens the link among raw-water variability, scaling risk, and long-term RO stability.

The integration of operational monitoring, membrane autopsy investigations, and CIP performance analysis provides a more comprehensive understanding of long-term reverse osmosis behavior under industrial operating conditions. Beyond a conventional water-quality assessment, the present study demonstrates that membrane fouling mechanisms remain a central challenge in maintaining stable desalination performance over time. The combination of optimized pretreatment, controlled chemical cleaning, and continuous operational monitoring contributed significantly to maintaining membrane efficiency despite high salinity and fluctuating raw-water quality conditions. These findings may support the future development of predictive maintenance and Digital Twin approaches for the intelligent supervision of industrial desalination systems.

## Figures and Tables

**Table 1:** Membrane operational parameters and fouling indicators used to assess long-term RO performance.

Operational Parameter	Source/Calculation Method	Operational Significance
Flux (L/m^2^·h)	SCADA: Permeate flow ÷ membrane area 22 L·m^−2^·h^−1^	Shows fouling-induced decline
TMP/Feed pressure	SCADA: 9.4–11.2 bar	Required for normalized permeability
Normalized permeability	Calculate: Flux ÷ TMP, temp-corrected	Industry standard fouling indicator
ΔP across stages	SCADA: 1.7 bar (1st), 2.3 bar (2nd)	Direct fouling indicator
SDI	SCADA shows 1.6	Pretreatment effectiveness
CIP frequency	1.5–4.5/month	Operational burden metric
SEC (kWh/m^3^)	Calculate from HP pump power	Economic/environmental impact
Antiscalant dosage	2.8 mg/L séquestrant	Scaling control verification

**Table 2:** Stationarity testing results (ADF and KPSS tests).

Parameter	n	ADF Statistic	ADF *p*-Value	KPSS Statistic	KPSS *p*-Value	Conclusion
Permeate Conductivity (µS/cm)	44	−2.55	0.1039	0.192	0.1000	Inconclusive
Feed Conductivity (µS/cm)	44	−2.31	0.1704	0.777	0.0100	Non-stationary
Calcium (mg/L)	43	−3.51	0.0078	0.515	0.0384	Inconclusive
Chloride (mg/L)	44	−2.80	0.0582	0.899	0.0100	Non-stationary
Salt Rejection (%)	44	−3.35	0.0129	0.057	0.1000	Stationary
Total Alkalinity (meq/L)	44	−15.55	<0.0001	0.319	0.1000	Stationary
Total Hardness (°F)	37	−3.84	0.0025	0.414	0.0713	Stationary

**Table 3:** Mann-Kendall trend test and Sen’s slope estimator results.

Parameter	n	S	Z	*p*-Value	Sen’s Slope (Monthly)	Sen’s Slope (Annual)	Trend
Permeate Conductivity (µS/cm)	44	112	1.12	0.2616	0.369	4.43	No trend
Feed Conductivity (µS/cm)	44	585	5.91	<0.0001	10.67	128.0	Increasing
Calcium (mg/L)	43	304	3.17	0.0015	0.315	3.77	Increasing
Chloride (mg/L)	44	539	5.44	<0.0001	2.82	33.85	Increasing
Salt Rejection (%)	44	84	0.839	0.4012	0.011	0.130	No trend
Total Alkalinity (meq/L)	44	−52	−0.516	0.6060	−0.0044	−0.050	No trend
Total Hardness (°F)	37	201	2.62	0.0089	0.060	0.720	Increasing

**Table 4:** Change point detection results (Pettitt and Buishand U tests). The asterisk (*) indicates the Buishand U statistic used to identify potential change points in the monitored time series.

Parameter	Pettitt CP Date	Pettitt U	Pettitt *p*-Value	Buishand CP Date	Buishand U *
Permeate Conductivity (µS/cm)	2021-06-01	194.0	0.1497	2021-07-01	7.33
Feed Conductivity (µS/cm)	2021-10-01	464.0	<0.0001	2021-10-01	17.19
Calcium (mg/L)	2022-05-01	327.0	0.0008	2022-05-01	11.91
Chloride (mg/L)	2022-01-01	401.0	<0.0001	2022-01-01	14.97
Salt Rejection (%)	2022-09-01	159.0	0.3506	2024-01-01	4.95
Total Alkalinity (meq/L)	2020-09-01	196.0	0.1419	2020-02-01	5.62
Total Hardness (°F)	2020-11-01	200.0	0.0198	2020-11-01	9.12

**Table 5:** Descriptive statistics of monitored parameters (2020–2024).

Parameter	n	Mean	Std Dev	CV (%)	Min	Max	Median	Q1	Q3
SDI	27	1.96	0.730	37.50	0.100	4.77	1.99	1.74	2.05
Reject Turbidity (NTU)	44	0.160	0.040	23.25	0.110	0.270	0.160	0.130	0.190
Reject Temperature (°C)	44	17.41	3.70	21.28	10.60	24.80	18.50	13.92	20.47
Feed Temperature (°C)	44	17.34	3.63	20.91	11.27	23.50	17.25	14.16	20.42
Permeate Conductivity (µS/cm)	44	170.8	31.60	18.50	114.7	295.0	166.8	153.5	188.7
Oxidability (mg O_2_/L)	43	1.46	0.270	18.26	1.10	2.80	1.44	1.37	1.48
Feed Turbidity (NTU)	44	0.120	0.020	17.92	0.090	0.180	0.110	0.100	0.120
Total Alkalinity (meq/L)	44	22.16	2.98	13.42	4.60	30.00	22.50	22.21	22.60
Calcium (mg/L)	43	100.7	10.14	10.07	77.00	131.2	100.8	95.22	105.0
Reject Conductivity (µS/cm)	44	10,807.7	992.1	9.18	9468.0	14,900.0	10,773.4	10,063.3	11,173.5
Chloride (mg/L)	44	760.8	51.53	6.77	652.3	847.5	765.9	731.5	797.2
Feed Conductivity (µS/cm)	44	2718.8	177.6	6.53	2325.0	3080.0	2724.5	2594.8	2852.3
Total Hardness (°F)	37	34.66	1.50	4.33	30.85	38.00	34.40	34.00	35.40
Permeate pH	44	6.10	0.100	1.71	5.88	6.46	6.10	6.04	6.15
Salt Rejection (%)	44	93.72	1.05	1.12	89.83	95.95	93.80	93.10	94.36
Reject pH	44	7.74	0.070	0.910	7.43	7.85	7.74	7.71	7.78

## Data Availability

The data presented in this study are available on reasonable request from the corresponding author. The data are not publicly available due to operational and institutional confidentiality restrictions.
